# Difficulty in Repurposing Selective Serotonin Reuptake Inhibitors and Other Antidepressants with Functional Inhibition of Acid Sphingomyelinase in COVID-19 Infection

**DOI:** 10.3389/fphar.2022.849095

**Published:** 2022-03-03

**Authors:** Pascal Le Corre, Gwenolé Loas

**Affiliations:** ^1^ Pôle Pharmacie, Service Hospitalo-Universitaire de Pharmacie, CHU de Rennes, Rennes, France; ^2^ Univ Rennes, CHU Rennes, Inserm, EHESP, Irset (Institut de Recherche en Santé, Environnement et Travail) - UMR_S 1085, Rennes, France; ^3^ Laboratoire de Biopharmacie et Pharmacie Clinique, Faculté de Pharmacie, Université de Rennes 1, Rennes, France; ^4^ Department of Psychiatry, Hôpital Erasme, Université Libre de Bruxelles (ULB), Brussels, Belgium; ^5^ Research Unit (ULB 266), Hôpital Erasme, Université Libre de Bruxelles (ULB), Brussels, Belgium

**Keywords:** selective serotonin reuptake inhibitors (SSRIs), functional inhibitors of acid sphingomyelinase (FIASMAs), COVID-19, fluvoxamine, repurposed drugs. (Min.5-Max. 8)

## Abstract

The rapid spread of COVID-19 has become a health emergency causing an urgent need for drug treatments to control the outbreak, especially in more vulnerable individuals. This is reinforced by the fact that prophylactic vaccines and neutralizing monoclonal antibodies may not be fully effective against emerging variants. Despite all efforts made by the scientific community, efficient therapeutic options currently remain scarce, either in the initial, as well as in the advanced forms of the disease. From retrospective observational studies and prospective clinical trials, selective serotonin reuptake inhibitors (SSRIs), and other antidepressants with functional inhibition of acid sphingomyelinase (FIASMAs), have emerged as potential treatments of COVID-19. This has led to some prematurely optimistic points of view, promoting a large prescription of fluvoxamine in patients with COVID-19, that we think should be reasonably tempered.

## Introduction

Many studies have proposed potential therapeutic approaches for treatment of COVID-19, especially for COVID-19 patients who are not hospitalized nor severely ill. Among these therapeutic options, antidepressants—especially selective serotonin reuptake inhibitors (SSRIs) - have emerged, being associated with protection against severe COVID-19.

It should be noted that several scientific journals, as well as mainstream press, have promoted the use of SSRIs, especially of fluvoxamine in patients with COVID-19 (Facente et al., 2021; Sidik 2021; Wroe et al., 2021). However, current evidence available in the literature, as well as some limitations in the studies performed, should invite caution in promoting a large prescription of fluvoxamine as a repurposed drug in the infectious phase in patients with COVID-19. However, as antidepressant drugs, SSRIs may be used in patients who have been infected with COVID-19 and may subsequently develop symptoms of depression.

## Current Evidence on Selective Serotonin Reuptake Inhibitors in Patients with COVID-19

Before the COVID-19 pandemic, numerous *in vitro* studies found that functional inhibitors of ASM (FIASMAs) disrupted infection by intracellular bacterial pathogens (Cockburn et al., 2019), Ebola virus (Johansen et al., 2015), severe acute respiratory syndrome coronavirus (SARS-CoV) (Dyall et al., 2014) or middle east respiratory syndrome coronavirus (MERS-CoV) (Dyall et al., 2014). Among FIASMAs with antiviral activity, SSRIs have been proposed as sertraline in Ebola virus disease (Johansen et al., 2015) or fluoxetine in influenza virus infection (Schloer et al., 2020), in hepatitis C virus (Young et al., 2014), coxsackievirus (Zuo et al., 2012), enterovirus (Bauer et al., 2019) and Ebola virus (Kummer et al., 2022). Beside antiviral properties, SSRIs have anti-inflammatory and immunomodulary properties that should be of value in the management of inflammatory lung disease in COVID-19 (Meikle et al., 2021; Pashaei 2021).

Retrospective observational studies in patients with COVID-19 have suggested that SSRIs (fluoxetine and fluvoxamine) may play a role in COVID-19 management. A retrospective study involving 345 patients with a medication order of antidepressants within 48 h of hospital admission has shown that antidepressant prescription was linked to a reduced likelihood of intubation or death, as a composite study endpoint (Hoertel et al., 2021a). More interestingly, a more powered retrospective observational study involving 3,401 patients with a medication order for a SSRI prescribed within a period ranging from 10 days before to 7 days after COVID-19 diagnosis, showed that SSRIs, and more specifically fluoxetine, have been associated with a reduced severity of COVID-19, as shown by a reduced relative risk of mortality (Oskotsky et al., 2021). Another retrospective study on 269 in hospitalized patients with pneumonia found a lower mortality rate in patients receiving fluoxetine (Németh et al., 2021). Two very recent retrospective observational studies on chronic treatment with antidepressants and notably SSRIs have given contradictory results. In a small size study in psychiatric inpatients, a significant association between antidepressant use and reduced COVID-19 infection was found notably for fluoxetine and trazodone (Clelland et al., 2021). The second study with a much higher number of subjects reported no significant difference in mortality between inpatients with COVID-19 receiving SSRIs and those not taking SSRIs (Rauchman et al., 2022). In the latter study, no patients were taking fluvoxamine.

These favorable effects of SSRIs have been linked to several potential underlying mechanisms of action including anti-inflammatory properties, agonist sigma-1 receptor (S1R) properties, as well as inhibition of acid sphingomyelinase/ceramide system. Within antidepressants with SIR agonist properties, fluvoxamine is the most powerful with a low nanomolar affinity (around 10-times more potent than fluoxetine), and early intervention of fluvoxamine (i.e., in the early infection and pulmonary stage) has been proposed (Hashimoto et al., 2022). Furthermore, taking into account that hypoxia could be a strong factor in COVID-19 progression, the use of fluvoxamine in the early phase of COVID-19 has been suggested in order to counteract some early posthypoxic events (Grieb et al., 2021; Grieb and Rejdak, 2021; Rejdak and Grieb, 2021). Additionally, retrospective observational studies have shown that calcium channel blockers (particularly amlodipine that is a FIASMA) promoting pulmonary vasodilatation, have improved clinical outcomes (Solaimanzadeh 2021).

Furthermore, functional inhibitors of acid sphingomyelinase (FIASMAs)—that include several SSRIs—were also associated to a reduced likelihood of intubation or death (Hoertel et al., 2021b). The role of FIASMAs *per se* in patients with COVID-19 has been previously highlighted (Schloer et al., 2020; Loas and Le Corre, 2021).

Beyond retrospective observational studies, prospective clinical trials with fluvoxamine - and to a lesser extent fluoxetine - have been set up: 3 trials are completed and the results have been published (Lenze et al., 2020; Reis et al., 2021; Seftel and Boulware, 2021), 5 studies are ongoing, 2 have been suspended, and 1 has been withdrawn. The main features of these studies are summarized in Table 1.

**TABLE 1:** Main features of completed, ongoing, suspended and withdrawn trial on fluvoxamine and fluoxetine in patients with COVID-19.

Trial identifier (ClinicalTrials.gov)	Sponsor - PI	Start date	Official title	Design	Participants planned	Drug studied (fluvoxamine/fluoxetine)	Dosing regimen	Study population and inclusion criteria	Endpoints	Main result	References
**Completed Trials**											
ND	Golden Gate Fields Medical Clinic, Berkeley, CA - D Seftel		ND	prospective open-label cohort	152 (74% completed the trial)	Fluvoxamine	50–100 mg (loading dose), followed by 50 mg twice daily for 14 days	SARS-COV-2 antigen positive patients, around 50% were asymptomatic - mean age was 42 years (interquartile range from 33 to 56 years)	rate of hospitalization	Incidence of hospitalization was zero % (0 of 65) in fluvoxamine group and 12.5% (6 of 48) in observation group. At 14 days, residual symptoms continued in zero % in fluvoxamine group and in 60% in observation group	Seftel and Boulware, (2021)
NCT04342663	Washington University School of Medicine - E Lenze	10 April 2020	A Double-blind, Placebo-controlled Clinical Trial of Fluvoxamine for Symptomatic Individuals With COVID-19 Infection	Randomized	152 (76% completed the trial)	Fluvoxamine	up to 300 mg daily as tolerated for around 15 days	Not hospitalized; recently tested SARS-CoV-2 (COVID-19 virus) positive. Currently symptomatic with one or more of the following symptoms (fever, cough, sore throat, mild dyspnea, diarrhea, vomiting, anosmia, ageusia, myalgia)	Number of participants who met clinical worsening (time frame: around 15 days) Clinical worsening defined as both of the following criteria: (a) dyspnea and/or hospitalization for shortness of breath or pneumonia, and (b) decrease in O2 saturation (<92%) and/or additionnal oxygen requirement in order to keep O2 saturation >92%	Clinical worsening reported in 0 of 80 patients in the fluvoxamine group, and in 6 of 72 patients in the placebo group (absolute difference, 8.7% [95% CI, 1.8–16.4%] from survival analysis; log-rank *p* = 0.009)	Lenze et al. (2020)
NCT04727424	Cardresearch - Brazil - G Reis	19 January 2021	TOGETHER trial - A Multicenter, Prospective, Adaptive, Double-blind, Randomized, Placebo-controlled Study to Evaluate the Effect of Fluvoxamine, Ivermectin, Doxasozin and Interferon Lambda 1A in Mild COVID-19 and High Risk of Complications	Multicenter, Prospective, Adaptive, Double-blind, Randomized, Placebo-controlled Study	1,506	Fluvoxamine, Ivermectin, Doxasozin and Interferon Lambda 1A	100 mg twice daily for 9 days	Acute flu-kike symptoms for less than 7 days. Patients with at least one enhancement criteria: Age >50 years; significant limitation of daily activities secondary to: dyspnea, chest pain myalgia, Patient with positive rapid test for SARS-CoV2 antigen performed at the screening or within 7 days of the beginning of symptoms. See the complete list of complications in the publication	composite endpoint of medical admission to a hospital setting resulting from:1) COVID-19- related illness defined as COVID-19 emergency setting visits with participants remaining under observation for >6 h or 2) referral for hospital isation secondary to progression of COVID-19 within 28 days of randomisation	In the fluvoxamine group 79 (11%) participants had a primary outcome event compared with 119 (16%) in the placebo group. Most events (87%) were hospitalisations	Reis et al. (2021)
** Trial Identifier (ClinicalTrials.gov)**	**Sponsor - PI**	**Start date**	**Official title**	**Design**	**Participants planned**	**Drug studied (fluvoxamine/fluoxetine)**	**Dosing regimen**	**Study population and inclusion criteria**	**Endpoints**	**Main result**	
**Ongoing Trials**											
NCT05087381	Chulalongkorn University - Thailand - DL Wannigama	october 1, 2021	Randomized-controlled Trial of the Effectiveness of COVID-19 Early Treatment in Community With Fluvoxamine, Bromhexine, Cyproheptadine, and Niclosamide in Decreasing Recovery Time	Open label, multiarm, prospective, adaptive platform, randomised controlled trial	1.800	Fluvoxamine, Bromhexine, Cyproheptadine, and Niclosamide	50 mg in the morning and 100 mg before bedtime, for a total of 14 days	COVID-19 patients with mild symptoms and confirmation by Antigen Test Kit or PCR for SARS-CoV-2. People who have symptoms consistent with COVID-19 and test positive for SARS-CoV-2 infection within 48 h of being known	Hospital admission or mortality related to COVID-19 [ Time Frame: Within 28 days ], Time taken to self- report recovery and Progression to severe COVID-19 disease		
NCT04885530	Duke University - S Naggie	8 June 2021	ACTIV-6: COVID-19 Outpatient Randomized Trial to Evaluate Efficacy of Repurposed Medications	Double-Blind, Placebo-Controlled, Randomized Trial	15.000	Ivermectin Fluvoxamine Fluticasone	50 mg twice a day for 10 days	Confirmed SARS-CoV-2 infection by any PCR or antigen test performed within 10 days of screening. Two or more symptoms of acute infection for ≤7 days. These symptoms are: fatigue, dyspnea, fever, cough, nausea, vomiting, diarrhea, body aches, chills, headache, sore throat, nasal symptoms, new loss of sense of taste or smell	Number of hospitalizations as measured by patient reports - Number of deaths as measured by patient reports - Number of symptoms as measured by patient reports [ Time Frame: Up to 14 days ]		
NCT04718480	SigmaDrugs Research Ltd., Hungary	27 November 2020	A Randomized, Double-blind, Placebo-controlled, Adaptive-design Study to Assess the Safety and Efficacy of Daily 200 mg Fluvoxamine as add-on Therapy to Standard of Care in Moderate Severity COVID-19 Patients	Randomized, double-blind, placebo-controlled, adaptive design add-on treatment study	100	Fluvoxamine	100 mg twice daily (with dose escalation and tapered dose reduction) for an overall treatment period of 74 days	Hospitalized patients with confirmed SARS-CoV-2 by PCR or known contact of confirmed case with syndrome consistent with coronavirus disease (COVID-19) with PCR pending (positive PCR result available prior to randomisation). Moderate cases (each of the followings symptoms met): dyspnoea but not manifest respiratory distress, respiratory rate 22-29/min; oxygen saturation at rest >93%; with or without the need for oxygen supplementation; pneumonia on medical imaging with pulmonary infiltrates occupying ≤50% of the lung-fields	Time to clinical recovery after treatment (time Frame: 74 days) - days from randomization (Day 1) to any 3 items within the following: resolution from fever (oral or tympanic temperature ≤37.5°C, axillary ≤37.0°C for at least 48 h without antipyretic drugs) return of respiratory rate to normal (≤20/min) normalization of SpO2 (≥95% on room air) cough remission (any reduction in cough-burden visual analogue scale, compared to Day 1)		
NCT04510194	University of Minnesota - C Bramante	1 January 2021	COVID-OUT: Early Outpatient Treatment for SARS-CoV-2 Infection (COVID-19)	Randomized	1.160	Metformin, fluvoxamine and ivermectin	50 mg twice daily for 14 days	Positive laboratory test for active SARS-CoV-2 viral infection based on local laboratory standard (i.e. +PCR) within 3 days of randomization. No known history of confirmed SARS-CoV-2 infection BMI≥25 kg/m2 by self-report height/weight or≥23 kg/m2 in patients who self-identify in South Asian or Latinx background. Willing and able to comply with study procedures (i.e., swallow pills) Has an address and electronic device for communication GFR>45 ml/min within 2 weeks for patients >75 years old, or with history of heart, kidney, or liver failure	Decreased oxygenation (Time Frame: 14 days) SpO2 =< 93% on home monitoring. Emergency Department Utilization (time Frame: 14 days) Emergency department utilization for Covid-19 Symptoms (CDC definition of Covid-19 symptoms)		
NCT04377308	University of Toledo Health Science Campus - C McCullumsmith	1 May 2020	Fluoxetine to Reduce Intubation and Death After COVID19 Infection	Non-Randomized	2.000	Fluoxetine	20–60 mg daily from 2 weeks to 2 months on symptom duration	COVID-19 test positive or presumptive positive awaiting COVID testing or results by following criteria: fever, cough and shortness of breath or presumptive positive by one of the 3 following criteria (fever, cough or shortness of breath) and known exposure to COVID-19 positive people within the past 14 days			
** Trial Identifier (ClinicalTrials.gov)**	**Sponsor - PI**	**Start date**	**Official title**	**Design**	**Participants planned**	**Drug studied (fluvoxamine/fluoxetine)**	**Dosing regimen**	**Study population and inclusion criteria**	**Endpoints**	**Main result**	
**Suspended trials**											
NCT04711863	Aisan Medical Center, Seoul, Korea - YP Chong	16 January 2021	Fluvoxamine for Adults With Mild to Moderate COVID-19: A Single-blind, Randomized, Placebo-controlled Trial	Randomized	400	Fluvoxamine	Up to 200 mg per day as tolerated until discharge from community treatment center or for approximately 10 days	Laboratory-confirmed SARS-CoV-2 patientswith. mild to moderate symptoms secondary to COVID-19 infection and admitted to community treatment centers. Symptoms consistent with COVID-19 with onset ≤7 days of randomization. Currently symptomatic with one or more of the following symptoms: fever, cough, myalgia, shortness of breath, nausea, anorexia, diarrhea, vomiting, anosmia, ageusia, sore throat, headache positive RT-PCR test for SARS-COV-2 infection ≤3 days of randomization	Clinical deterioration (time Frame up to 10 days) Defined as one of the following symptoms: 1) decreased O2 saturation (SpO2 <94%) on room air, 2) Supplemental oxygen requirement to maintain O2 saturation ≥94%, 3) deterioratio of pneumonia with dyspnea: clinically worsening condition estimated by clinician with increased infiltration of chest X-ray or minute respiratory rate over 25.4) WHO Clinical Progression Scale ≥5 including intubation and death)		
NCT04668950	Washington University School of Medicine - E Lenze	22 December 2020	Fluvoxamine for Early Treatment of Covid-19: a Fully-remote, Randomized Placebo Controlled Trial	Randomized	683	Fluvoxamine	up to 200 mg per day as tolerated, for approximately 15 days	Not hospitalized SARS-CoV-2 positive (laboratory test or by physician report). Currently symptomatic with one or more of the following symptoms (fever, cough, myalgia, mild dyspnea, chest pain, diarrhea, nausea, vomiting, anosmia, ageusia, sore throat, or nasal congestion)	Clinical deterioration [ Time Frame: RCT-approximately 15 days ] Defined as both of the following: 1)Presence of dyspnea and/or hospitalization for shortness of breath or pneumonia, 2)) decrease in O2 saturation (<92% on room air) and/or supplemental oxygen requirement to keep O2 saturation ≥92%)	By May 2021, STOP COVID 2 stopped enrolling new participants based on an overall lower rate of clinical deterioration than expected	
**Withdrawn trials**											
NCT04570449	Milton S. Hershey Medical Center - E Saunders	November 2020	Fluoxetine to Reduce Hospitalization From COVID-19 Infection (FloR COVID-19)	Randomized	40 (zero recruited)	Fluoxetine	fluoxetine 20 mg daily for 8 weeks according the following schedule: week 1 = 20 mg, week 2 = 40 mg, weeks 3–6 = 60 mg, week 7 = 40 mg, week 8 = 20 mg	Tested positive for active SARS-CoV-2 infection and less than 10 days since first appearance of symptoms; Fever persists >24 h without the use of fever reducing medications; and presence other symptoms of COVID-19 as described by the CDC	Rate of hospitalization (time frame: 8 weeks) Measures number of subjects hospitalized for COVID-19 symptoms Physical symptoms assessed via daily checklist (time frame: 8 weeks)		

Among the currently published studies, the study by Lenze (Lenze et al., 2020) has shown a lower probability of clinical deterioration within 15 days in the fluvoxamine group. In this first study performed in a rather small number of patients (80 in the treatment group), fluvoxamine was dosed 100 mg three-times daily from randomization up to 15 days. The second study by Reis (Reis et al., 2021) has shown a risk reduction of 5% (32% relative risk reduction) on the primary outcome of hospitalization. This outcome was defined as either a retention in a COVID-19 emergency setting or a transfer to tertiary hospital attributable to COVID-19. In this second and larger trial (741 in the treatment group), fluvoxamine was dosed 100 mg twice daily for 9 days.

It should be noted that a larger trial (STOP COVID 2) of fluvoxamine (expected 683 participants given fluvoxamine up to 200 mg per day for 15 days) has stopped enrolling new participants as of May 2021 due to futility and as a result, shows that a lower than expected rate of clinical deterioration may have needed a higher number of participants (Table 1).

Based on the available literature, the the IDSA guideline panel recommendation 24 (IDSA guideline, 2021) panel recommendation 24 indicates that among ambulatory patients with COVID-19, fluvoxamine should be used only within clinical trials to better delineate the effects of fluvoxamine on disease progression, such as need for hospital admission, ICU care, and ultimately mortality (Infectious Diseases Society of America Guidelines on the Treatment and Management of Patients with COVID-19, version V5.5.3 updated 9 November 2021).

Furthermore, there are currently 2 meta-analysis in a preprint state (Kacimi et al., 2021, submitted 2021; Lee et al., 2021, submitted 2021) and it should be noted that they did not include the same studies, only 2 studies in common on the 3 included studies. A meta-analysis has concluded that fluvoxamine has not shown significant efficacy in reducing either the mortality rates or mechanical ventilation while promising results in decreasing the likelihood for hospitalization in patients with COVID-19 (Kacimi et al., 2021, submitted 2021). The second meta-analysis (including unpublished data from the suspended trial STOP COVID 2) has concluded that fluvoxamine showed a high probability of preventing hospitalization in outpatients with COVID-19, and that this drug could be a treatment option for patients providing the lack of contraindication (Lee et al., 2021, submitted 2021).

## Discussion

We would like to point out three important methodological limitations of the current larger study by Reis (Reis et al., 2021) related to the primary endpoint, the absence of control in affectivity, and to the fluvoxamine dosing regimen that deserved discussion. These comments might also help to temper the conclusions of the two meta-analysis.

First, the use of a composite criterion constitutes a strong limitation of this study. The reasons for the retention in an emergency setting, and for the transfer to a tertiary hospital are not documented, and the likelihood for implication of non-COVID-19 urgency reasons cannot be ruled out. Moreover, when differences between groups were examined for each component of the criterion, only the retention in emergency setting reached statistical significance (7/741 vs 36/756 in fluvoxamine and placebo groups, respectively). Indeed, the difference in hospitalization, that represented 87% of the total events, was not significant (75/741–10% vs 97/756–13% in fluvoxamine and placebo groups, respectively). Thus, the difference in the composite criterion is mainly explained by the difference in the rate of retention in the emergency setting. The mortality rate was not significantly different between the two groups (fluvoxamine: 17/741, 2% vs placebo: 25/756, 3%, *p* = 0.24). Considering the rate of mortality reported (2 and 3% in the fluvoxamine and placebo groups, respectively), a number of subjects of 3,788 by group would have been required to reach the 33% difference observed in the study (80% power, α level of 0.05, bilateral test). Hence, the promising or the high probability of preventing hospitalization in outpatients as suggested by the meta-analysis should be reasonably ruled out.

Second, an important confounding factor has not been considered. The level of positive and/or negative effects has not been controlled in the Together trial (Reis et al., 2021) contrary to the previous trial on fluvoxamine by Lenze (Lenze et al., 2020) that reported comparable levels of anxiety in the two groups since anxiety may relate to shortness of breath. Furthermore, fluvoxamine is an antidepressant and anxiolytic drug which will increase pain thresholds (Palmer and Benfield, 1994). Hence, subjects with low negative affect and/or increased pain threshold may be less aware of their symptoms and therefore less prone to go an emergency setting.

Third, the effect of fluvoxamine should occur with some delay as the elimination half-life (T1/2) is 14–22 h, and is increased by 30–50% after multiple dosing (van Harten 1993). Hence, steady-state concentrations are reached within 5–10 days after initiation of therapy, and even beyond 10 days in the elderly since T1/2 is doubled. In the Reis study, time to emergency setting visit and time to hospitalization were 4 and 5 days, respectively. Thus, the duration of treatment in the study may not be suitable to establish a link between fluvoxamine and the primary endpoint. This pharmacokinetic feature also applies to retrospective observational studies. A study has shown that the administration of a low dose (37.5 mg) of amitriptyline in healthy subjects was sufficient to reduce ASM activity *in vitro*, and to prevent *ex vivo* the infection of freshly isolated nasal epithelial cell (Carpinteiro et al., 2020). The *ex vivo* protective effect of amitriptyline against infection appeared within a short delay (1.5 h), and lasted for at least 24 h. The study suggested that low dosage of amitriptyline could prevent infection of freshly isolated nasal epithelial cells with SARS-Cov-2 and with a very short time of action.

Considering that clinical symptoms of COVID-19 affecting notably the lungs, antidepressant concentration in this tissue would be an important factor to consider. A pre-print study (Eugene 2022) simulated the tissue concentrations of fluoxetine and estimated the percentage of patients achieving a trough level for the effective concentration of lungs (based on a 90% inhibition of SARS-CoV-2 as reported in Calu-3 human lung cells). At a median daily dose of 40 mg of fluoxetine, inhibiting concentrations in the lung would be achieved in 60% of patient at day 1, 90% at day 5 and 93% at day 10 of dosing. However, it should be noticed that considering a mean elimination half-life of 3.5 days of fluoxetine reported in the study (quite smaller than that observed in patients, around 4–6 days), the simulated plasma profile is quite unusual since 50% of the steady-state should be reached in 1 half-life (3.5 days). This was not the case in the simulation where around 75% of the steady-state was reached within one half-life suggesting an overestimation of the initial plasma and corresponding tissue concentrations so that the percentage of patients with effective concentration of lungs and day 1 may be overestimated.

Apart from methodological caveats, we think that the promotion for a widespread use of fluvoxamine is currently inappropriate, and that other intrinsic pharmacological properties of the drug should be considered that have been underlined by several authors (Glebov, 2021). Although the adverse events during the clinical trials (Lenze et al., 2020; Reis et al., 2021) on fluvoxamine have been moderate, the incidence of clinically significant drug-drug interactions or of psychiatric adverse reactions of SSRI (e.g., insomnia, anxiety, …) could be a concern on a large-scale use (Blaess et al., 2021). Furthermore, fluvoxamine is a not a drug with smart pharmacokinetics. Fluvoxamine has a high first-pass metabolism and displays a non-linear and sex-dependent pharmacokinetics with a high inter-patient variability (Altamura et al., 2015). Fluvoxamine is metabolized by a CYP2D6 that displays a marked racial/ethnic difference in frequency of functional alleles. Moreover, fluvoxamine is a potent inhibitor of CYP1A2, and a moderate inhibitor of CYP3A4, CYP2C9 and CYP2C19 so it may lead to drug-drug interactions with the numerous drugs prescribed in chronic diseases (17.4% of the case of severe adverse events reported to the FAERS are related to drug interaction, FDA Adverse Event Reporting System, consulted December 31, 2021). Thus, prescription of fluvoxamine to patients with medical comorbidities should be considered with caution.

We do hope that the ongoing trials will help to refineg the place for SSRIs in the treatment of COVID-19 in its initial stages, including their safety. This may come from the results of ACTIV-6 and of COVID-OUT that are enrolling a significant number of patients with different arms exploring not only fluvoxamine but also other drugs (Table 1). However, within these trials, a non-randomized study with fluoxetine will not help to better understand the positioning of this SSRI. This is unfortunate since the retrospective observational study by Oskotsky (Oskotsky et al., 2021) has suggested a positive impact of fluoxetine on the risk of mortality.

However, the role of SSRIs and other antidepressants may better be considered in advanced forms of COVID-19. Indeed, some of these drugs have FIASMAs properties (Loas and Le Corre, 2021); and there is preclinical evidence that ASM has an essential role in the pathogenesis of sepsis/host response, and that its inhibition might have a positive influence on the outcome (Chung and Claus, 2021). Furthermore, activation of the sphingomyelinase-ceramide pathway in response to infection in intensive care patients with severe COVID-19 has shown a potential benefit in the inhibition of ASM (Abusukhun et al., 2021). Additionally, a small-sized open-label study of COVID-19 ICU hospitalized patients (prospective cohort trial with matched controls in patients, *n* = 51 with a follow up of 15 days), showed that fluvoxamine (100 mg three-times daily) reduced the overall mortality 58.8 vs 76.5%) (Calusic et al., 2021).

Targeting both the host and the virus should also be considered given the synergy of the combination of fluoxetine with direct-antiviral agents (e.g., remdesivir and its metabolite GS-441524) that has been displayed on *ex vivo* cellular models (Brunotte et al., 2021; Schloer et al., 2021). Moreover, the combination of a host-directed drug (i.e., baricitinib) and of an antiviral (i.e., remdesivir) in patients hospitalized with moderate to severe disease for COVID-19 has been proven beneficial in reducing the recovery time, and in speeding up the improvement in clinical outcomes (Kalil et al., 2021). Hence, a more systematic study of the combination of SSRIs with direct-antiviral agents should be of value. However, a parallel estimation of the potential *in vivo* drug-drug interactions between these drugs (both as victim drug and as perpetrator) should be carried out given the potential for drug-drug interactions of SSRI’s (e.g., fluoxetine and fluvoxamine) and of some antivirals. Remdesivir and molnupiravir are pro-drugs metabolized by non-CYP450 enzymes with short half-lives and are less prone to drug-drug interactions, and thus the evaluation of DDI should consider their active moiety. However, the recently marketed ritonavir-boosted association with nirmatrelvir (Paxlovid™) should be used with caution with regard to drug-drug interactions in COVID-19 as a result of the well known DDI potential of ritonavir (Heskin et al., 2022).

## Conclusion and Future Directions

It is clear that positioning SSRIs in outpatients with COVID-19 is not that simple and that methodological precautions have to be considered. The use of the DREL scale (i.e., drug repositioning evidence level, Oprea and Overington, 2015) may help assess of the quality of the scientific evidence from drug repositioning studies. Given the time-course of COVID-19 symptoms with a rapid deterioration in high-risk patients, the ideal drug should have either a short elimination half-life or should be started with a loading dose to rapidly reach the steady state. Beyond the daily dose, the dosing regimen (i.e., interval between doses) should also be considered since the interval between the doses influences the accumulation ratio as well as the fluctuations in blood and tissue levels that may be of concern. Drug safety profile is also an issue, and non-antidepressants FIASMAs having fewer side effects and already widely prescribed in the population, such as melatonin or antihistamines, may be an option.

However, the benefit-risk balance of SSRIs, and of other antidepressants especially with FIASMA properties, in treating advanced forms COVID-19 in hospitalized patients is much more favorable and should be considered and investigated. In these patients, combined strategies with host-directed drugs and direct-antiviral agents are also an issue to consider. In this perspective, a drug such as amitriptyline that showed improvement in the early pro-inflammatory response associated with sepsis in pre-clinical models (Xia et al., 2019a; Xia et al., 2019b; Chung and Claus, 2021), as well as a potential to prevent infection of isolated human nasal epithelial cells with COVID-19 (Carpinteiro et al., 2020; Loas and Le Corre, 2021) may represent an option for clinical trials in this setting.

## Data Availability

The original contributions presented in the study are included in the article/Supplementary Material, further inquiries can be directed to the corresponding author.
